# UV-Vis spectroscopy coupled with firefly algorithm-enhanced artificial neural networks for the determination of propranolol, rosuvastatin, and valsartan in ternary mixtures

**DOI:** 10.1038/s41598-025-89187-7

**Published:** 2025-03-29

**Authors:** Ahmed Serag, Maram H. Abduljabbar, Yusuf S. Althobaiti, Farooq M. Almutairi, Shaker T. Alsharif, Rami M. Alzhrani, Marwa F. Ahmed, Atiah H. Almalki

**Affiliations:** 1 Pharmaceutical Analytical Chemistry Department, Faculty of Pharmacy, Al-Azhar University, Nasr City, Cairo 11751 Egypt; 2 Department of Pharmacology and Toxicology, College of Pharmacy, Taif University, P.O. Box 11099, 21944 Taif, Saudi Arabia; 3 Addiction and Neuroscience Research Unit, Health Science Campus, Taif University, P.O. Box 11099, 21944 Taif, Saudi Arabia; 4 Department of Clinical Laboratories Sciences, College of Applied Medical Sciences, University of Hafr AlBatin, 39524 Hafr AlBatin, Saudi Arabia; 5 Department of Pharmaceutical Sciences, College of Pharmacy, Umm Al-Qura University, Makkah, Saudi Arabia; 6 Department of Pharmaceutics and Industrial Pharmacy, College of Pharmacy, Taif University, P.O. Box 11099, 21944 Taif, Saudi Arabia; 7 Department of Pharmaceutical Chemistry, College of Pharmacy, Taif University, P.O. Box 11099, 21944 Taif, Saudi Arabia

**Keywords:** Artificial neural networks, Cardiovascular diseases, Firefly algorithm, Greenness assessment, Spectrophotometry, Analytical chemistry, Spectrophotometry

## Abstract

In the present study, a simple, rapid and cost-effective analytical method was developed for the simultaneous determination of three commonly prescribed cardiovascular drugs: propranolol, rosuvastatin and valsartan. The method employed artificial neural networks (ANN) to model the relation between the UV absorption spectra of the drugs and their concentrations. An experimental design of 25 samples was employed as a calibration set, and a central composite design of 20 samples was used as a validation set. The firefly algorithm (FA) was evaluated as a variable selection procedure to optimize the developed ANN models resulting in simpler models with improved predictive performance as evident by lower relative root mean square error of prediction (RRMSEP) values compared to the full spectrum ANN models. Validation of the developed FA-ANN models demonstrated excellent accuracy, precision and selectivity for the quantification of the target analytes as per international conference on harmonisation (ICH) guidelines. Additionally, the greenness, analytical practicality and sustainability of the developed models were assessed using the analytical greenness (AGREE), blue applicability grade index (BAGI) and the red-green-blue (RGB) tools, confirming their environmentally friendly, practical and sustainable nature. This research shed the light on the potential of ANN coupled with UV fingerprinting for the rapid and simultaneous determination of critical cardiovascular drugs posing a significant impact on pharmaceutical quality control and patient monitoring.

**Supplementary Information:**

The online version contains supplementary material available at 10.1038/s41598-025-89187-7.

## Introduction

Cardiovascular diseases (CVDs) are major health concerns worldwide, encompassing a broad spectrum of conditions affecting the heart and blood vessels, including coronary artery disease, hypertension, heart failure, and stroke^1^. In 2020, it was estimated that 19 million deaths were related to CVDs, reflecting a 32% increase compared to the previous decade^2^. The multifaceted nature of CVDs often necessitates a comprehensive therapeutic approach, employing various drug classes to target different pathophysiological mechanisms^3^. These three drugs, propranolol, rosuvastatin, and valsartan, are commonly prescribed pharmaceutical agents that demonstrate this multifaceted approach to treatment^4–6^. Propranolol, a non-selective beta-blocker, acts by competitively inhibiting the effects of catecholamines on β-adrenergic receptors, thereby reducing heart rate, myocardial contractility, and blood pressure^7^. This makes it particularly effective in managing hypertension, angina, and certain arrhythmias^8^. Rosuvastatin, a potent β-Hydroxy β-methylglutaryl-CoA (HMG-CoA) reductase inhibitor, plays a crucial role in lipid management by reducing cholesterol synthesis in the liver, leading to increased low-density lipoprotein (LDL) receptor expression and enhanced clearance of circulating LDL-cholesterol^9,10^. Valsartan, an angiotensin II receptor blocker, selectively inhibits the binding of angiotensin II to the AT1 receptor, resulting in vasodilation, reduced aldosterone secretion, and improved cardiac function^11^. It is particularly beneficial in treating hypertension, heart failure, and diabetic nephropathy. The combination of these drugs addresses multiple risk factors and pathways involved in CVDs, offering synergistic effects that can significantly improve patient outcomes. Furthermore, combination therapy often allows for lower doses of individual drugs, potentially reducing side effects while maintaining or even enhancing therapeutic efficacy.

The accurate and reliable quantification of these cardiovascular drugs is crucial for ensuring appropriate dosing, monitoring treatment efficacy, and optimizing patient care^12^. High-performance liquid chromatography (HPLC) remains the gold standard for pharmaceutical analysis^13–19^, offering high selectivity and sensitivity through various detection modes. Reversed-phase HPLC with UV detection has been widely employed, typically achieving limits of detection in the nanogram range, with both isocratic and gradient elution modes being utilized depending on sample complexity. The technique’s robustness, precision, and broad linear dynamic range have made it particularly suitable for quality control applications. However, HPLC methods often require extensive method development, complex mobile phase compositions, and significant organic solvent consumption.

Liquid chromatography-mass spectrometry (LC-MS)^20–23^ has emerged as a powerful complementary technique, providing enhanced selectivity and sensitivity particularly valuable for bioanalytical applications and impurity profiling. The technique’s ability to provide structural information and achieve detection limits in the picogram range has made it indispensable for certain applications. Despite these advantages, LC-MS systems are expensive to acquire and maintain, require highly trained operators, and often involve complex sample preparation protocols including solid-phase extraction or liquid-liquid extraction steps.

Traditional chromatographic methods often face the challenge of addressing the growing environmental concerns associated with the pharmaceutical industry^24^. These methods typically involve significant organic solvent consumption, high energy requirements for instrument operation, and substantial waste generation from mobile phases and sample preparation steps. This has led to an increasing interest in the development of more sustainable, “green” analytical techniques that minimize the use of hazardous solvents and reagents, reduce energy consumption, and generate less waste. UV spectroscopy, a simple, rapid, and relatively inexpensive analytical tool, has been explored as a potential alternative for the determination of these cardiovascular drugs^25^. While offering advantages in terms of simplicity, speed, and environmental impact, conventional UV spectroscopic methods face limitations in resolving complex mixtures due to spectral overlap, highlighting the need for advanced data analysis approaches^26^. Machine learning algorithms, such as Artificial neural networks (ANN), have emerged as powerful tools for the analysis of complex spectroscopic data, demonstrating the ability to model both linear and non-linear relations and extract relevant chemical information from UV fingerprints^27,28^. For example, ANN has been successfully applied to the quantification of amoxicillin and flucloxacillin in their pharmaceutical formulations using UV spectroscopy^27^. Moreover, approaches like the use of variable selection techniques such as genetic and firefly algorithms have been employed to further enhance the performance of these ANN-based UV spectroscopic methods^29^.

The present study aimed to develop a simple method based on ANN as multivariate calibration models for the simultaneous quantification of propranolol, rosuvastatin, and valsartan, three commonly prescribed cardiovascular drugs, utilizing their characteristic UV fingerprints. A design of experiment approach was employed to generate the calibration and validation data sets. The influence of the Firefly Algorithm as a variable selection procedure on the developed ANN models was also evaluated. Additionally, the models were validated using an external validation set and according to ICH guidelines to ensure their analytical reliability. The developed FA-ANN models were then applied to determine the content of these drugs in their commercial pharmaceutical formulations. Additionally, the greenness, blueness and whiteness of the developed models have been assessed to determine their environmental impact, analytical practicality and sustainability, posing the current approach as a rapid, cost-effective and environmentally friendly alternative to conventional analytical methods.

## Experimental

### Materials and reagents

Propranolol hydrochloride, rosuvastatin calcium, and valsartan were obtained from were obtained from the Egyptian Drug Authority (EDA) with purities exceeding 98%. Pharmaceutical formulations containing the three drugs as their active ingredients including (Inderal^®^ claimed to contain 10 mg of propranolol HCl per tablet, Crestor^®^ claimed to contain 10 mg of rosuvastatin calcium per tablet and Diovan^®^ claimed to contain 80 mg of valsartan per tablet) were purchased from local pharmacies in Cairo, Egypt. Distilled water was used throughout the study.

### Instrumentation

A Shimadzu UV-1800 spectrophotometer equipped with 1 cm quartz cells was used to record the UV absorption spectra of the drug solutions in the range of 200–400 nm. Scan speed was set to fast with 1 nm interval to obtain the UV fingerprints of the studied analytes. Data processing was performed using UV Probe software (version 2.43).

### Standard solutions

Stock solutions of propranolol hydrochloride (100 µg/mL), rosuvastatin calcium (100 µg/mL), and valsartan (100 µg/mL) were prepared separately by dissolving 10 mg of each drug in 100 mL of distilled water to obtain concentrations of 100 µg/mL.

### Procedures

#### Calibration and validation data set

A partial factorial design of experiments was employed to generate the calibration data set, involving 3 factors at 5 levels each, resulting in 25 samples (Table S1). The chosen factors were the concentrations of the three drugs, with the central level set at 6 µg/mL and the lower and upper levels at 2 and 10 µg/mL, respectively. This concentration range was selected to ensure the results were within the linear dynamic range of the proposed UV spectroscopic method. For the validation set, a central composite design of 20 samples was used to evaluate the predictive ability of the developed ANN models over a wider range, providing a more robust assessment of their performance (Table S2). To improve the statistical significance of the validation results, axial points with alpha = 1.5 and center point replicates were included.

#### ANN models development

The UV absorption spectra of the calibration and validation sample sets were recorded and used as the input variables for the ANN models under MATLAB^®^ R2016a environment. Initially, regions above 350 nm were excluded from the input data as they exhibited low absorbance that might limit the models’ predictive ability. The backpropagation algorithm was employed to train the ANN models using the UV fingerprints as inputs and the known each drug concentrations as outputs. The ANN architecture was optimized by varying the number of hidden layers and neurons to obtain the best predictive performance using relative root mean square error of cross validation (RRMSECV) as the performance criterion for model optimization. Furthermore, the Firefly algorithm, a nature-inspired meta-heuristic optimization technique, was investigated as a variable selection tool to identify the most informative wavelengths for the development of the ANN models. Several key validation figures of merit were calculated, including relative root mean square error of prediction (RRMSEP) and the coefficient of determination, to evaluate the predictive accuracy and robustness of the developed ANN models.

Additionally, accuracy and precision were evaluated as per international conference on harmonisation (ICH) guidelines via determination of the% recovery and relative standard deviation (RSD%), respectively, for different samples analyzed in the same day and in three consecutive days. Selectivity was also assessed by standard addition techniques to determine the matrix effect and evaluate the ability of the model to quantify each drug in the presence of the sample matrix.

#### Application to the pharmaceutical samples

To demonstrate the practical applicability of the optimized FA-ANN models, they were employed for the determination of propranolol, rosuvastatin and valsartan in their pharmaceutical formulations. To prepare the sample solutions, an accurately weighed amount of each tablet corresponding to 10 mg of each drug was dissolved in 100 mL of distilled water, sonicated for 15 min, filtered through 0.45 μm syringe filters, and diluted to the appropriate concentration range with distilled water before recording the UV spectra. The content of each drug in the pharmaceutical preparations was calculated using the developed FA-ANN models and the results were compared to those obtained by the reference HPLC methods^14,17,18^. The reference HPLC methods employed different chromatographic conditions: propranolol was analyzed using a C18 column (4.6 × 100 mm, 2.5 μm) with a mobile phase consisting of methanol:0.1% orthophosphoric acid (75:25, v/v) at a flow rate of 1.0 mL/min and detection at 257 nm^14^. For rosuvastatin, separation was achieved on a ZORBAX Eclipse C18 column (4.6 × 150 mm, 5 μm) using a gradient elution of acidified water and acetonitrile with detection at 291 nm^17^. Valsartan analysis was performed using a green HPLC method with a mobile phase consisting of 0.02 M ammonium acetate (pH 7.2) and ethanol in gradient mode^18^. These validated reference methods provided a reliable basis for comparing the performance of our developed FA-ANN models.

### Greenness, blueness and whiteness assessment

The proposed analytical method was evaluated in terms of its greenness, blueness, and whiteness using the analytical greenness (AGREE)^30^ and blue applicability grade index (BAGI)^31^ and red-green-blue (RGB12)^32^ tools, respectively. The greenness assessment AGREE considered parameters such as waste generation, energy consumption, and the use of hazardous chemicals. This tool can be downloaded from https://mostwiedzy.pl/AGREE. The choice of the AGREE parameters was selected based on the principles of green chemistry and the specific requirements of the analytical method. The blue applicability grade index assesses the analytical practicality of the method, including considerations such as sample throughput, time, and cost. The BAGI tool is publicly available at https://mostwiedzy.pl/en/justyna-plotka-wasylka,647762-1/BAGI. The whiteness assessment was also performed using the RGB tool, which evaluates the overall analytical performance, environmental impact and analytical feasibility of the method. The excel file used for the RGB assessment is available as supplementary file in^32^.

## Results and discussion

### Spectral characteristics

**Fig. 1:**
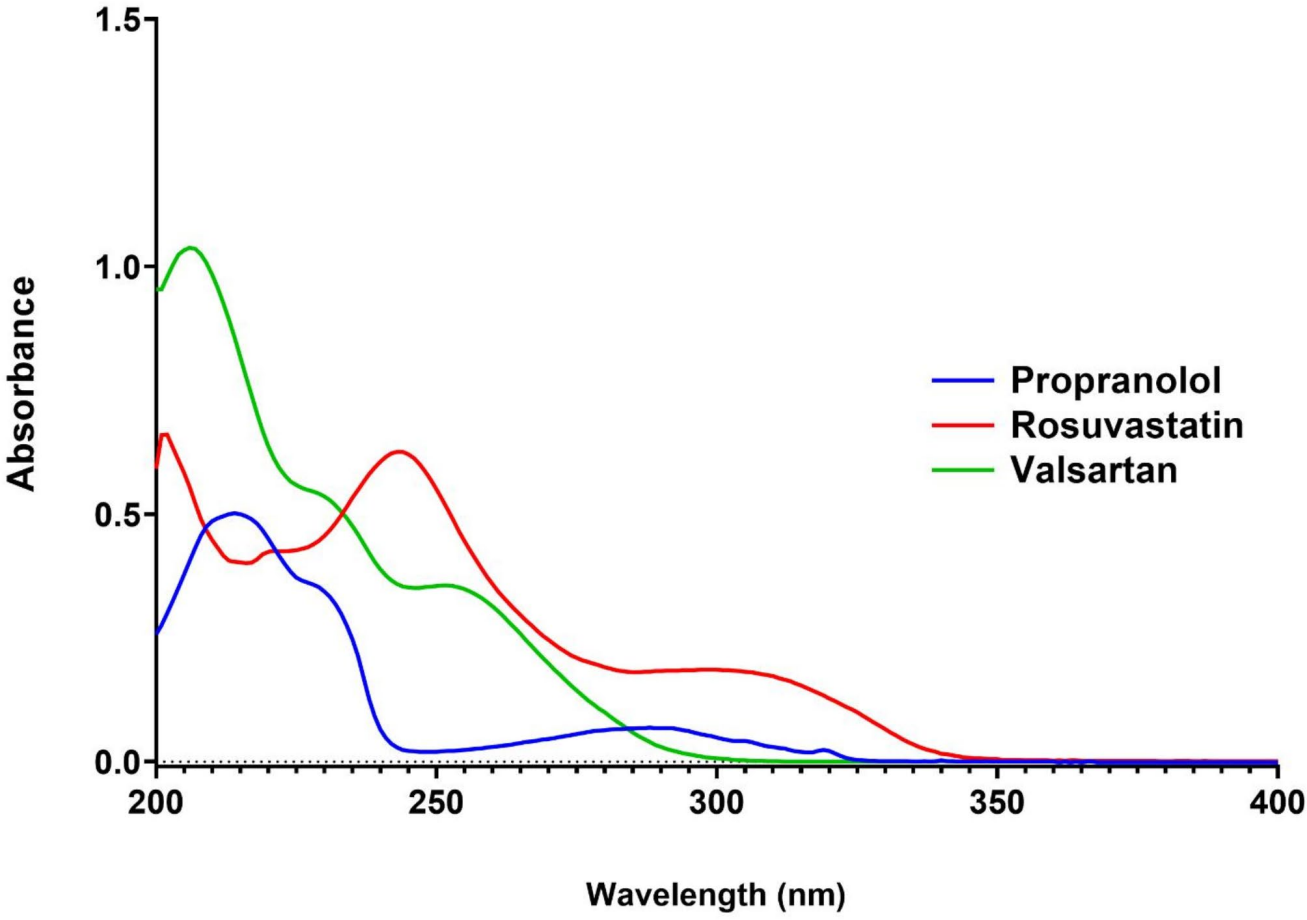
Zero order absorption spectra of propranolol, rosuvastatin and valsartan showing sever overlap hindering their direct determination in their ternary mixture.

The UV absorption spectra of the three drugs exhibited distinct absorption bands in the 200–350 nm region, with noticeable differences in their absorption intensities and wavelengths of maximum absorption (λ_max_) (Fig. 1). Specifically, propranolol showed a characteristic peak at 214 nm, rosuvastatin at 243 nm, and valsartan at 246 nm. Such close spectral overlapping emphasized the need for employing advanced multivariate techniques, such as ANN, to resolve the individual contributions of each drug in the mixture.

### Models’ optimization and development

**Fig. 2:**
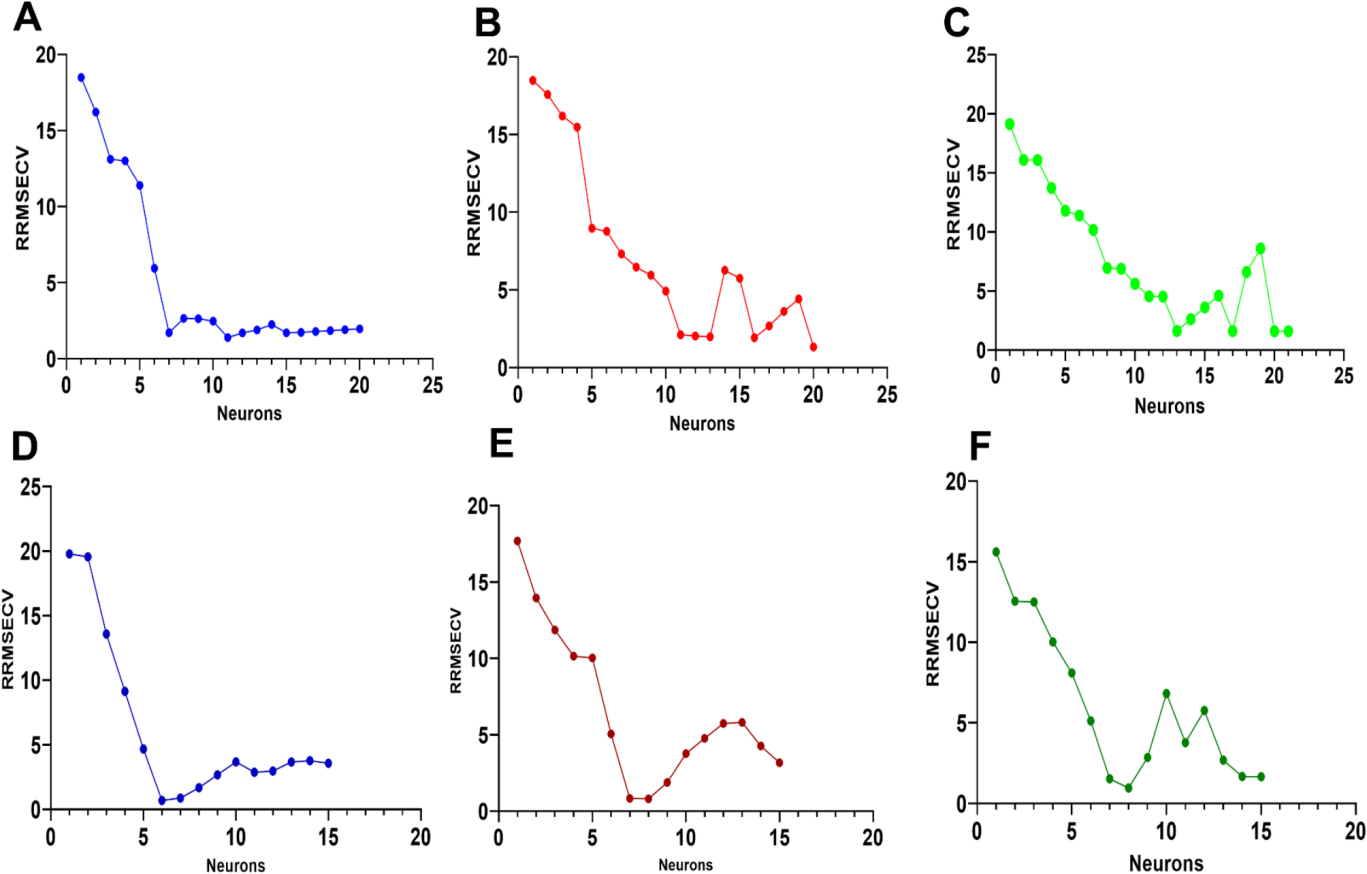
Cross-validation results using leave-one-out procedures showing RRMSECV values versus number of neurons for both model types: Full ANN models for (**A**) propranolol, (**B**) rosuvastatin, and (**C**) valsartan; and FA-ANN models for (**D**) propranolol, (**E**) rosuvastatin, and (**F**) valsartan. The plots demonstrate significant decreases in RRMSECV values at the optimum number of neurons for each model.

The first step in the ANN model development process was to optimize the network architecture by varying the number of hidden layers and neurons to achieve the best predictive performance. The input data was the 151 wavelength values of the calibration set UV spectra, while the output layer consisted of 1 neuron representing the concentrations of each drug. Back propagation algorithm was utilized for training the ANN models, and the RRMSECV was used as the performance criterion. The critical step is to optimize the number of hidden layers and neurons to avoid any overfitting of the data. Previous studies have shown that a single hidden layer ANN is generally adequate for resolving binary and ternary mixtures. Consequently, a single hidden layer ANN architecture was employed in this work, with the number of neurons varied from 1 to 20. RRMSCV values were calculated for each ANN architecture, and the optimal number was calculated based on the lowest RRMSCV that satisfied the model stability and good predictive ability. It was found that 7 neurons in the hidden layer provided the best compromise between model complexity and predictive accuracy for propranolol determination with RRMSCV of 1.72% (Fig. 2A). On the other hand, 11 and 13 neurons were selected for rosuvastatin and valsartan ANN models with RRMSCV of 2.11% and 1.62%, respectively (Fig. 2B, C).

**Fig. 3:**
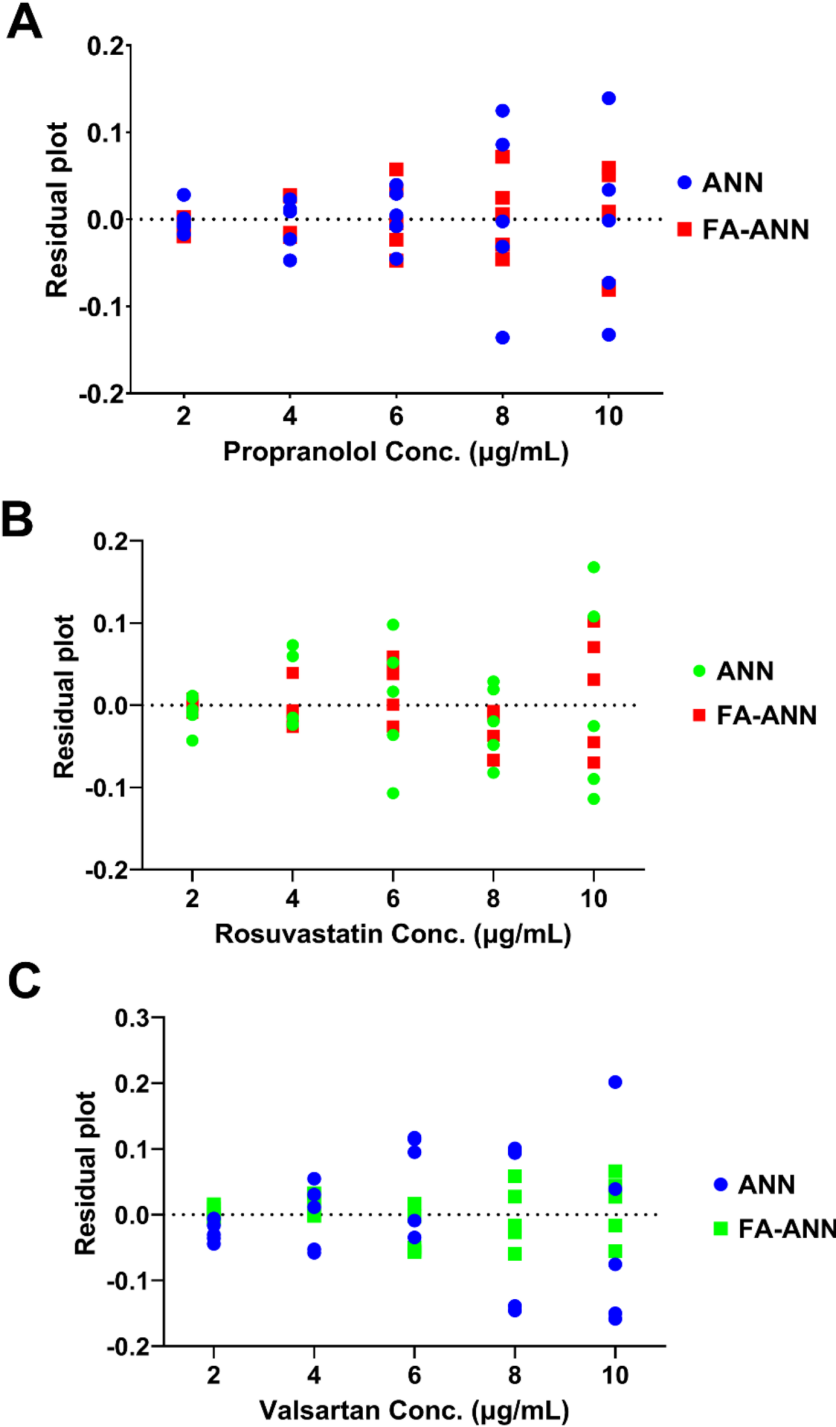
Residual concentration plots of the calibration set as calculated using the developed ANN and FA-ANN models for (**A**) propranolol, (**B**) rosuvastatin and (**C**) valsartan.

To further enhance the predictive performance, the firefly algorithm was employed as a wavelength selection tool. The algorithm mimics the flashing behavior of fireflies to identify the most influential wavelengths that maximize the ANN model’s predictive ability. The optimization process started with a population of random fireflies (solutions), where each firefly’s position was encoded as a binary string (‘1’ for selected wavelengths, ‘0’ for excluded wavelengths), allowing thorough exploration of the solution space. Through iterative optimization, the algorithm improved these solutions based on firefly brightness, which was determined by a fitness function using RRMSECV as the optimization criterion. Brighter fireflies (solutions with lower RRMSECV) attracted dimmer ones, moving the population toward optimal wavelength combinations. Several parameters affecting the Firefly algorithm’s performance, such as the number of fireflies, maximum generation, light absorption coefficient (γ), attraction coefficient (βο) and randomization parameter (α), were optimized through a grid search procedure (Table S3). The process continued until convergence, defined as no significant improvement in RRMSECV for 50 consecutive iterations. This procedure allowed the identification of the most important wavelengths that exhibited the highest sensitivity towards the drug concentrations, resulting in reduced input dimensionality to 47 wavelengths for propranolol (Fig. S1A), 43 wavelengths for rosuvastatin (Fig. S1B), and 40 wavelengths for valsartan (Fig. S1C). The developed FA-ANN models using the selected wavelengths exhibited significantly improved predictive performance compared to the initial full spectrum ANN models, with even a reduced number of neurons for all three drugs and lower RRMSCV values of 0.68% for propranolol (Fig. 2D), 0.84% for rosuvastatin (Fig. 2E), and 0.096% for valsartan (Fig. 2F). Additionally, further optimization of all hyperparameters, including learning rate, transfer function, and learning function, was performed to ensure the robustness of the final models (Table 1). Among these hyperparameters, the learning rate exhibited different optimal values for each drug e.g. 0.1 for propranolol, 10 for rosuvastatin, and 100 for valsartan. Such higher values for rosuvastatin and valsartan models could be attributed to the specific characteristics of the Levenberg–Marquardt (TRAINLM) algorithm, which balances gradient descent and Gauss–Newton steps, allowing for stable convergence even with larger values. Additionally, the FA-based variable selection reduced the dimensionality of the spectral data, simplifying the ANN models and enhancing their training efficiency. This resulted in faster convergence during ANN training and enabled the models to tolerate higher learning rates. Examination of the residual plots for the actual vs. predicted values for the calibration sets further confirmed the excellent fit and predictive performance of the models (Fig. 3). Notably, the FA-ANN models demonstrated superior prediction accuracy compared to standard ANN models, as evidenced by the distribution of residuals around the zero line. For all three drugs, FA-ANN models produced more compact residual distributions, particularly at higher concentrations where standard ANN models showed greater dispersion. This improved clustering of residuals around zero indicates enhanced prediction consistency and reduced model bias. Based on these findings, the developed FA-ANN models were selected as the optimal ANN architectures for further evaluation and application.

**Table 1:** Optimized parameters of FA-ANN for propranolol, rosuvastatin and valsartan.

Drug	Propranolol	Rosuvastatin	Valsartan
Architecture	47-6-1	43-7-1	40-8-1
Hidden neurons number	6	7	8
Transfer functions	Purelin–Purelin		
Learning rate	0.1	10	100
Training function	TRAINLM		

### Models’ validation and figures of merit

**Fig. 4:**
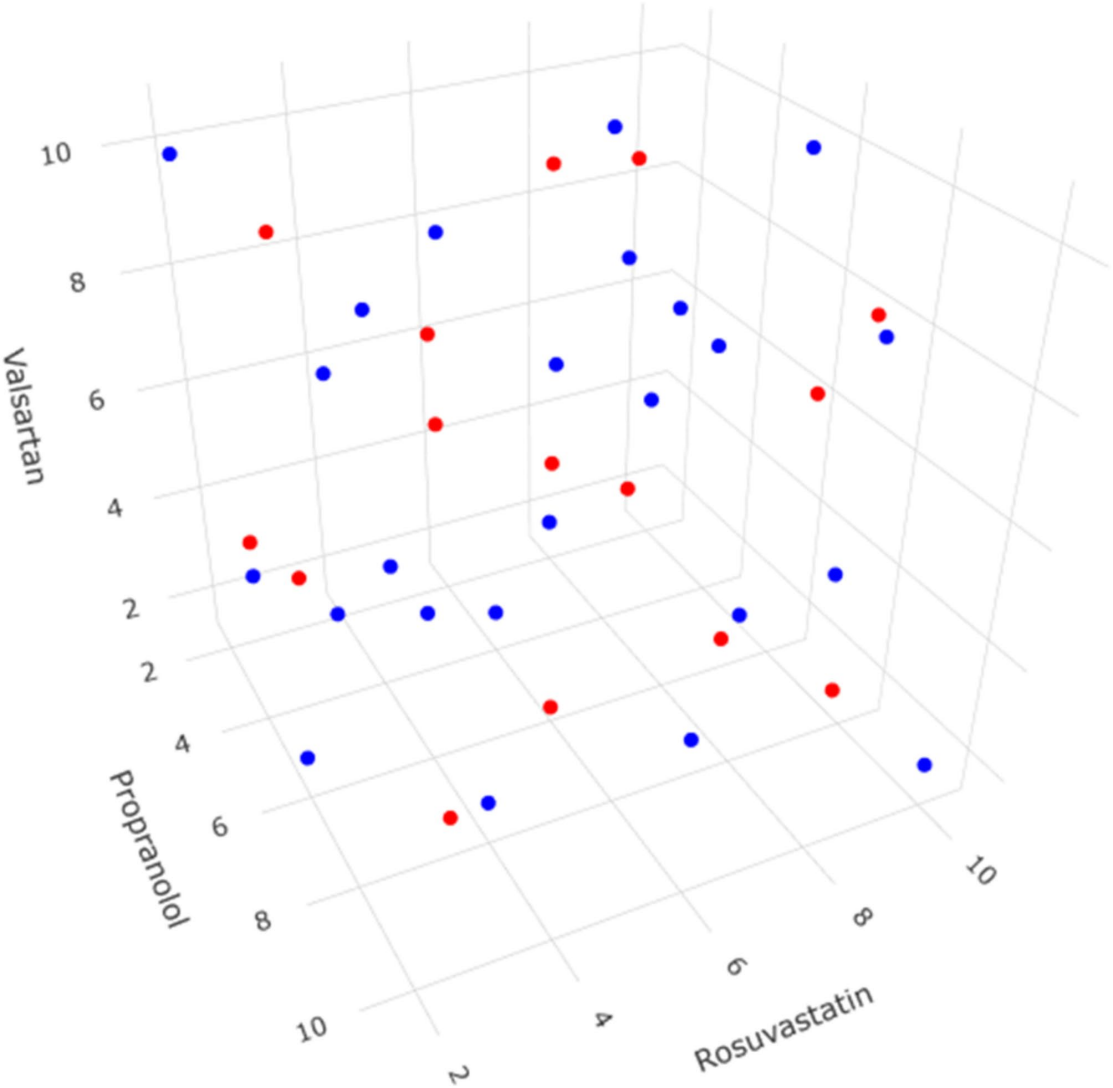
The 3D experimental space showing the positioning of the calibration and validation samples for propranolol, rosuvastatin and valsartan. Blue dots represent the calibration set while the red dots represent the validation set.

To assess the robustness and predictive ability of the optimized FA-ANN models, a central composite design validation set comprising 20 samples was employed (Fig. 4). The validation set samples were prepared independently and their UV spectra were recorded. The predicted concentrations from the FA-ANN models were plotted against the actual concentrations, and the coefficient of determination (R^2^) was calculated to evaluate the goodness-of-fit. The results showed excellent linearity with R^2^ values of 0.9998, 0.9997, and 0.9998 for propranolol, rosuvastatin, and valsartan, respectively, with intercept values close to zero and slopes close to unity, indicating negligible bias and high predictive ability of the developed models (Table 2). In addition, other figures of merit such as RRMSEC, RRMSEP, and BCRRMSEP were calculated to further evaluate the models’ performance. These results demonstrated the excellent predictive ability and robustness of the optimized FA-ANN models (Table 2).

**Table 2:** Validation results of propranolol, rosuvastatin and valsartan by the proposed FA-ANN models.

Drug	Slope^a^	Intercept^a^	*R* ^2a^	LOD (µg/mL)	LOQ (µg/mL)	RRMSEC	RRMSEP	RBCMSEP
Propranolol	0.9999	0.0006	0.9998	0.1340	0.4062	0.6633	1.2528	0.0942
Rosuvastatin	1.0012	− 0.0031	0.9997	0.1413	0.4282	0.7024	1.1879	0.0847
Valsartan	0.9955	0.0173	0.9998	0.1260	0.3819	0.6455	1.2964	0.1008

^a^Data obtained by plotting the actual versus the predicted values.

Furthermore, accuracy of the developed models was assessed by recovery studies as per ICH guidelines. The results showed excellent accuracy with recovery values ranging from 99.15 to 101.83% for all three drugs confirming the suitability of the proposed method for their quantitative analysis (Table S4). Intra-day and inter-day precision were also evaluated, and the low RSD% values (< 2%) indicated the high precision of the method (Table S4). Besides, selectivity of the developed models was confirmed by standard addition technique where known amounts of the drugs were spiked into pharmaceutical formulations, and the recoveries were found to be satisfactory with values in the range of 98.18–101.83% (Table S5).

### Application to pharmaceutical samples

The optimized and validated FA-ANN models were applied for the determination of propranolol, rosuvastatin, and valsartan in various pharmaceutical formulations such as Inderal^®^ (propranolol) and Crestor^®^ (rosuvastatin) and Diovan^®^ (valsartan) tablets. The results were in good agreement with the labeled amounts, demonstrating the applicability of the developed method for routine quality control analysis with recovery values ranging from 98.94 to 101.78% (Table 3). The results were compared statistically with reference HPLC methods^14,17,18^ for each drug in terms of mean and variance, and no significant differences were found (P values > 0.05) (Table 3), further confirming the reliability and accuracy of the proposed UV-Vis spectroscopic method coupled with FA-ANN for the determination of these drugs in pharmaceutical preparations.

**Table 3:** Quantitative analysis of propranolol, rosuvastatin and valsartan in commercial pharmaceutical preparations by the proposed methods and statistical comparison with the reported methods.

Drug	Method	Mean^a^	SD	Variance	t-test (2.306)^b^	*P* value	F-value (6.338) ^b^	*P* value
Propranolol	FA-ANN	100.63	2.114	4.468	0.767	0.466	1.549	0.682
	Reported method^14^	99.70	1.698	2.884				
Rosuvastatin	FA-ANN	100.13	1.184	1.403	0.912	0.395	3.009	0.311
	Reported method^17^	99.16	2.055	4.222				
Valsartan	FA-ANN	100.99	1.71	2.923	1.315	0.226	1.402	0.751
	Reported method^18^	99.67	1.444	2.085				

^a^Average of five determinations.

^b^The values in parenthesis are tabulated values of “*t* “and “*F*”.

### Greenness, blueness and whiteness assessments

Initial evaluation of the environmental impact of the developed FA-ANN models-based UV-Vis method revealed its benign nature compared to traditional HPLC techniques. Water was used as the only solvent, thus avoiding the use of volatile organic solvents and their associated environmental and health concerns. UV-Vis spectrometry also requires lower sample and reagent volumes compared to HPLC, further reducing the environmental impact. In terms of energy consumption, the instrumentation used in this method is less energy intensive than HPLC. Waste generation is also significantly lower with the proposed approach with only minimal amounts of aqueous solutions that can be easily disposed of. All these initial investigations verify the greenness of the proposed method.

**Fig. 5:**
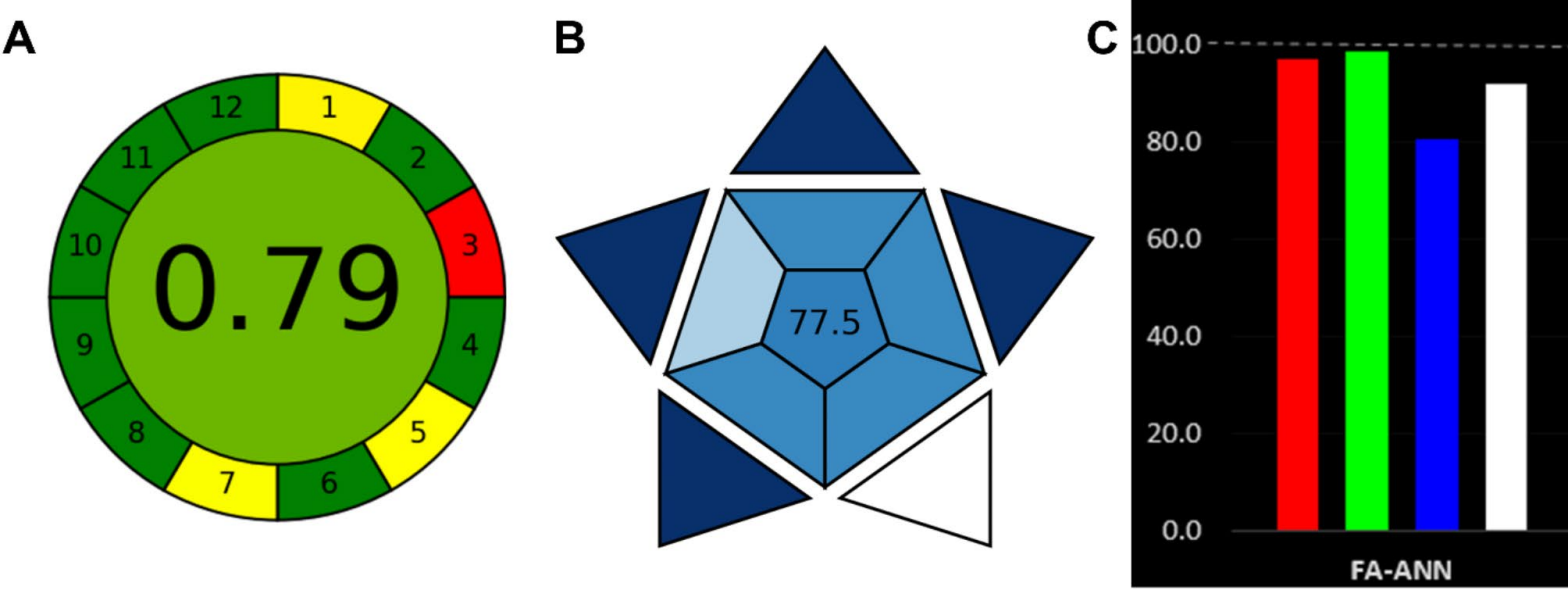
(**A**) Greenness assessment of the developed FA-ANN method using the AGREE tool to determine its environmental impact. (**B**) Blueness assessment of the developed FA-ANN method using the BAGI tool to determine its analytical practicability. (**C**) Whiteness assessment of the developed FA-ANN method using the RGB12 algorithm to determine its sustainability.

However, a more comprehensive assessment of the greenness of the developed models was carried out using the AGREE tool, which takes into account various parameters such as reagent consumption, waste generation, energy use, and health/environmental impacts in a quantitative manner based on the 12 Principles of Green Chemistry. The AGREE score calculated for the UV-Vis FA-ANN method was 0.79 (Fig. 5A), indicating a high level of greenness. The major contributors to the high greenness score were the use of water as the only solvent, minimized sample and reagent volumes, and lower energy consumption compared to HPLC. However, factors related to the sampling procedure and position of analytical device as well as degree of automation could be further optimized to enhance the overall greenness of the proposed approach.

Additionally, the blueness of the developed models in terms of their analytical practicality was evaluated using the BAGI tool. This tool considers factors such as sample preparation, analysis time, analysis cost, and equipment requirements. The BAGI score calculated for the UV-Vis FA-ANN method was 77.5 (Fig. 5B), indicating a high level of analytical practicality. The major contributors to the high blueness score were the simple sample preparation, rapid analysis time, and low analysis cost posing the current method as a viable alternative to traditional HPLC for routine quality control analysis of the target drugs in pharmaceutical formulations and aid in the application of machine learning algorithms in pharmaceutical analysis.

Furthermore, the RGB12 algorithm was employed to assess the overall whiteness of the proposed method, taking into account the analytical performance (red), environmental impact (green) and the practical nature of the developed FA-ANN models (blue). An overall whiteness score of 92.1 was obtained (Fig. 5C), indicating the high quality and desirability of the method. High red and green scores were obtained due to the excellent analytical performance and environmental friendliness of the method, while a slightly lower blue score was attributed to the need for further optimization of the sampling and automation aspects. Such comprehensive greenness, blueness and whiteness assessments demonstrate the superior analytical characteristics, environmental compatibility, and practical applicability of the proposed UV-Vis FA-ANN method for the determination of propranolol, rosuvastatin and valsartan in pharmaceutical formulations.

## Conclusion and future perspectives

In this work, a novel analytical methodology integrating UV-Vis spectroscopy with FA-ANN modeling was successfully developed and validated for the simultaneous determination of propranolol, rosuvastatin, and valsartan in pharmaceutical formulations. The FA-ANN methodology demonstrated high analytical performance with reduced RRMSCV values (0.68%, 0.84%, and 0.096% for propranolol, rosuvastatin, and valsartan, respectively), excellent linearity (R^2^ > 0.999), and reliable accuracy (98.94–101.78% recovery). The FA optimization significantly enhanced method efficiency by reducing spectral inputs by approximately 70% while maintaining robust performance. Environmental and practical assessments validated the method’s viability through high AGREE (0.79), BAGI (77.5), and whiteness (92.1) scores, confirming its potential as a green analytical alternative to traditional HPLC methods.

However, several limitations must be acknowledged, including the need for strict control of spectroscopic conditions, manual sampling procedures, and potential matrix effects in complex samples. Future research should focus on extending this methodology to other drug combinations, validating performance in biological matrices, and developing automated sampling procedures. Additionally, exploring alternative variable selection techniques and hybrid approaches combining UV-Vis with other analytical techniques could further enhance the method’s capabilities. The successful implementation of this FA-ANN methodology provides a basis for future developments in sustainable pharmaceutical analysis, offering a practical pathway toward greener analytical chemistry practices in quality control laboratories.

## Electronic supplementary material

Below is the link to the electronic supplementary material.

Supplementary Material 1

## Data Availability

The data presented in this study are available on request from the corresponding author.
