# Supplementary material for: UV-Vis spectroscopy coupled with firefly algorithm-enhanced artificial neural networks for the determination of propranolol, rosuvastatin, and valsartan in ternary mixtures
Source: Sci Rep. 2025 Mar 29;15:10838. doi: 10.1038/s41598-025-89187-7 (PMC11953262; doi:10.1038/s41598-025-89187-7)
Supplement: Supplementary file 1 — Supplementary Material 1 [file 41598_2025_89187_MOESM1_ESM.docx]

**UV-Vis Spectroscopy Coupled with Firefly Algorithm-Enhanced Artificial Neural Networks for the Determination of Propranolol, Rosuvastatin, and Valsartan in Ternary Mixtures**

**Ahmed Serag** ^a, *^**, Maram H Abduljabbar** ^b^**, Yusuf S. Althobaiti** ^b, c^, **Farooq M. Almutairi** ^d^**, Shaker T.** **Alsharif** ^e^**, Rami M. Alzhrani** ^f^, **Marwa F. Ahmed** ^g^**, Atiah H. Almalki** ^c, g^

^a^ Pharmaceutical Analytical Chemistry Department, Faculty of Pharmacy, Al-Azhar University, Nasr City 11751, Cairo, Egypt

^b^ Department of Pharmacology and Toxicology, College of Pharmacy, Taif University, P.O. Box 11099, Taif 21944, Saudi Arabia

^c^ Addiction and Neuroscience Research Unit, Health Science Campus, Taif University, P.O. Box 11099, Taif 21944, Saudi Arabia

^d^ Department of Clinical Laboratories Sciences, College of Applied Medical Sciences, University of Hafr AlBatin, Hafr AlBatin 39524, Saudi Arabia

^e^ Department of Pharmaceutical Sciences, College of Pharmacy, Umm Al-Qura University, Makkah, Saudi Arabia

^f^ Department of Pharmaceutics and Industrial Pharmacy, College of Pharmacy, Taif University, P.O. Box 11099, Taif 21944, Saudi Arabia

^g^ Department of Pharmaceutical Chemistry, College of Pharmacy, Taif University, P.O. Box 11099, Taif 21944, Saudi Arabia

*Corresponding author email address: **(Ahmed Serag**) [Ahmedserag777@azhar.edueg](mailto:Ahmedserag777@azhar.edueg)

**Table S1:** The concentrations of the calibration set as calculated *via* implementing a partial factorial experimental design approach.

| No. | Propranolol (µg/mL) | Rosuvastatin (µg/mL) | Valsartan (µg/mL) |
| --- | --- | --- | --- |
| 1 | 6 | 6 | 6 |
| 2 | 6 | 2 | 2 |
| 3 | 2 | 2 | 10 |
| 4 | 2 | 10 | 4 |
| 5 | 10 | 4 | 10 |
| 6 | 4 | 10 | 6 |
| 7 | 10 | 6 | 4 |
| 8 | 6 | 4 | 4 |
| 9 | 4 | 4 | 8 |
| 10 | 4 | 8 | 10 |
| 11 | 8 | 10 | 8 |
| 12 | 10 | 8 | 6 |
| 13 | 8 | 6 | 10 |
| 14 | 6 | 10 | 10 |
| 15 | 10 | 10 | 2 |
| 16 | 10 | 2 | 8 |
| 17 | 2 | 8 | 2 |
| 18 | 8 | 2 | 6 |
| 19 | 2 | 6 | 8 |
| 20 | 6 | 8 | 8 |
| 21 | 8 | 8 | 4 |
| 22 | 8 | 4 | 2 |
| 23 | 4 | 2 | 4 |
| 24 | 2 | 4 | 6 |
| 25 | 4 | 6 | 2 |

**Table S2:** The concentrations of the validation set as calculated *via* implementing a central composite experimental design approach with 5 central points.

| No. | Propranolol (µg/mL) | Rosuvastatin (µg/mL) | Valsartan (µg/mL) |
| --- | --- | --- | --- |
| 1 | 9 | 9 | 3 |
| 2 | 9 | 9 | 9 |
| 3 | 3 | 3 | 3 |
| 4 | 6 | 6 | 6 |
| 5 | 3 | 9 | 9 |
| 6 | 3 | 9 | 3 |
| 7 | 6 | 6 | 6 |
| 8 | 6 | 6 | 6 |
| 9 | 3 | 3 | 9 |
| 10 | 9 | 3 | 3 |
| 11 | 9 | 3 | 9 |
| 12 | 6 | 6 | 6 |
| 13 | 6 | 6 | 1.5 |
| 14 | 6 | 6 | 6 |
| 15 | 1.5 | 6 | 6 |
| 16 | 6 | 6 | 10.5 |
| 17 | 6 | 10.5 | 6 |
| 18 | 10.5 | 6 | 6 |
| 19 | 6 | 6 | 6 |
| 20 | 6 | 1.5 | 6 |

**Table S3:** The optimized parameters of the Firefly algorithm as a variable selection procedure to enhance the ANN models' predictability.

| Parameter | Propranolol | Rosuvastatin | Valsartan |
| --- | --- | --- | --- |
| Number of fireflies | 48 | 40 | 56 |
| Maximum generations | 400 | 300 | 700 |
| α | 0.15 | 0.2 | 0.3 |
| β_ο_ | 1.3 | 1.5 | 1.4 |
| γ | 1 | | |

**Table S4:** Accuracy and precision results for the determination of propranolol, rosuvastatin and valsartan by the proposed FA-ANN method.

| *Concentration (ng/mL)* | | | *Accuracy*  *(% R ± SD)* [*^a^*](https://www.sciencedirect.com/science/article/pii/S1386142524003305#tblfn1) | | | | *Precision (RSD%)* [*^a^*](https://www.sciencedirect.com/science/article/pii/S1386142524003305#tblfn1) | | | | | |
| --- | --- | --- | --- | --- | --- | --- | --- | --- | --- | --- | --- | --- |
| Propranolol (µg/mL) | Rosuvastatin (µg/mL) | Valsartan (µg/mL) | Propranolol | Rosuvastatin | Valsartan | Propranolol | | | Rosuvastatin | | Valsartan | |
|  |  |  |  |  |  | *Intra-day* | | *Inter-day* | *Intra-day* | *Inter-day* | *Intra-day* | *Inter-day* |
| 4 | 6 | 8 | 99.15  ±  0.897 | 101.76  ±  1.412 | 100.21  ±  1.225 | 0.86 | | 1.605 | 0.934 | 1.618 | 0.257 | 1.342 |
| 5 | 5 | 5 | 101.15  ±  0.734 | 98.18  ±  1.254 | 99.83  ±  1.267 | 0.975 | | 1.73 | 0.338 | 1.332 | 0.556 | 1.531 |
| 6 | 4 | 4 | 99.64  ±  0.962 | 100.11  ±  1.212 | 101.83  ±  1.135 | 0.127 | | 1.187 | 0.45 | 1.166 | 0.555 | 1.801 |
| 8 | 8 | 6 | 101.53  ±  1.464 | 101.57  ±  1.495 | 99.81  ±  1.175 | 0.626 | | 1.94 | 0.355 | 1.492 | 0.506 | 0.811 |

[^a^](https://www.sciencedirect.com/science/article/pii/S1386142524003305#tblfn1) Average of three determinations

| Drug | | Pharmaceutical taken (µg/mL) | | Pharmaceutical found (µg/mL) | Pure added (μg/mL) | Pure found (µg/mL) | %R |
| --- | --- | --- | --- | --- | --- | --- | --- |
| Propranolol | | 4.0 | | 4.025 | 2 | 1.983 | 99.15 |
|  |  |  |  |  | 3 | 3.035 | 101.15 |
|  |  |  |  |  | 4 | 3.986 | 99.64 |
| Mean ± %RSD | | | | | | | 99.98±1.043 |
| Rosuvastatin | | 4.0 | | 3.966 | 2 | 2.031 | 101.53 |
|  |  |  |  |  | 3 | 3.053 | 101.76 |
|  |  |  |  |  | 4 | 3.927 | 98.18 |
| Mean ± %RSD | | | | | | | 100.49±1.994 |
| Valsartan | 4.0 | | 4.040 | | 2 | 2.004 | 100.21 |
|  |  |  |  |  | 3 | 2.995 | 99.83 |
|  |  |  |  |  | 4 | 4.073 | 101.83 |
| Mean ± %RSD | | | | | | | 100.62±1.056 |

**Table S5:** Selectivity study of the proposed method using standard addition technique.


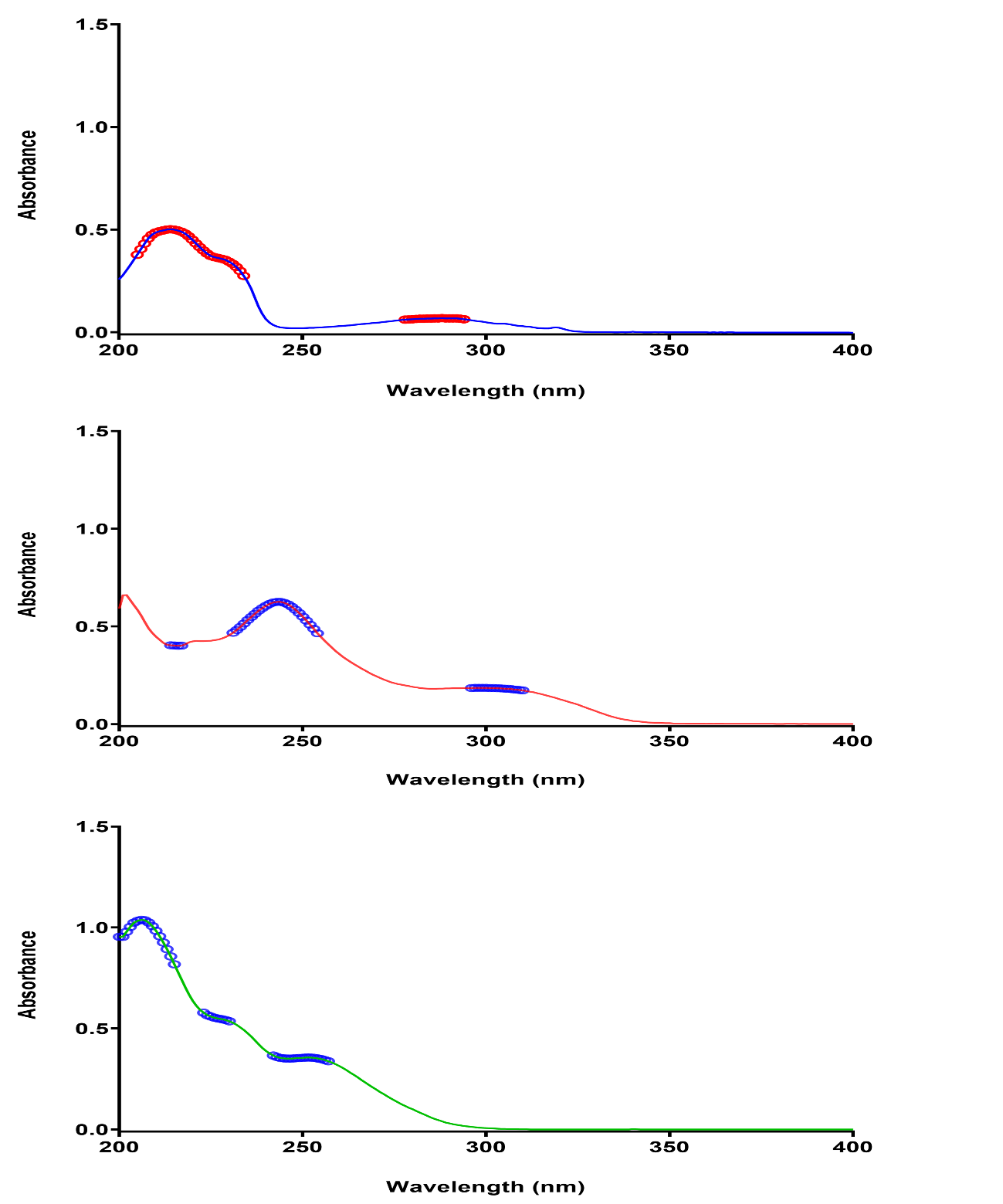


**Fig. S1:** UV absorption spectra and wavelengths selected by the Firefly Algorithm for (A) propranolol, (B) rosuvastatin, and (C) valsartan in the range of 200-400 nm. The selected wavelengths represent the optimal regions identified by FA for each drug's quantification in the developed ANN models.
